# From probability to plausibility in climate-risk scenarios: a cognitive framework for decision-making under deep uncertainty

**DOI:** 10.3389/fcogn.2026.1928401

**Published:** 2026-08-13

**Authors:** Jeffery R. L. Pendleton, Paul W. Pritchard, David Wilkinson, Clive Wilkins, Nicola S. Clayton

**Affiliations:** 1 Department of Psychology, University of Cambridge, Cambridge, United Kingdom; 2 Cambridge Institute for Sustainability Leadership, University of Cambridge, Cambridge, United Kingdom; 3 UK Centre for Greening Finance and Investment, University of Oxford, Oxford, United Kingdom

**Keywords:** climate-risk scenarios, constructive memory, institutional decision-making, mental time travel, radical uncertainty, scenario planning, simulational fluency, episodic memory

## Abstract

Climate-risk scenario analysis has become central to financial supervision, adaptation planning, and institutional governance, yet formally modeled futures often fail to guide action. We argue that the gap between representation and response is partly cognitive. Drawing on constructive memory, mental time travel, source monitoring, episodic future thinking, scenario planning, and organizational sensemaking, we develop a framework of plausible futures for decision-making under deep uncertainty. On this account, climate futures gain practical force when they become mentally constructible, narratively scaffolded, socially stabilized, and culturally legitimated. Simulational fluency is proposed as one pathway through which scenario form may influence how readily decision-makers construct, inspect, compare, and rehearse possible futures. The framework identifies false precision in quantitative models and the ways narrative can sharpen or mislead judgement. These insights support the joint development of qualitative and quantitative approaches. Climate-risk governance requires sophisticated calculation together with anticipatory practices that allow institutions to inhabit uncertain futures cognitively; those practices keep uncertainty explicit and judgement both revisable and answerable to evidence.

## 1 Why climate futures fail to govern action

Climate change is one of the most extensively modeled risks facing contemporary institutions, yet modeled climate futures do not automatically become action-guiding. Financial supervisors, firms, governments, and adaptation planners increasingly rely on scenarios to reason about long horizons, nonlinear change, physical risk, transition risk, and systemic disruption (Bank of England, 2024; Bank for International Settlements, 2023; Bolton et al., 2020; Carney, 2015). A central problem concerns the usability of increasingly sophisticated representations and whether those futures can inform judgement and circulate through organizational deliberation sufficiently to affect present action. Climate change exposes the limits of the assumption that better representation will by itself generate better response, because the difficulty extends from model production into the cognitive and institutional processes through which futures acquire meaning and force (Chenet et al., 2021; Kiesel and Stahl, 2023; Lawrence et al., 2020).

Deep uncertainty unsettles the assumption that better data and more sophisticated models will, by themselves, improve climate-risk judgement. Nonlinear change, path dependence, tipping dynamics, spatial unevenness, and decades-long horizons can make future outcomes hard to picture concretely and hard to hold within shared institutional understanding (Lawrence et al., 2020; Paddeu and Lyons, 2024). Climate-related financial risk illustrates the point. Disclosure-led approaches remain constrained by radical uncertainty. Conventional risk-management frameworks confront model uncertainty and broader systemic uncertainty in settings where stable historical analogs are absent (Chenet et al., 2021; Kiesel and Stahl, 2023). A developing body of finance and risk literature further indicates that increasing model complexity can create false precision and narrow the recognized range of futures. Plausible harms may then remain underappreciated under severe uncertainty (Aikman et al., 2021; Bradley, 2025; Shortridge and Guikema, 2017).

From that starting point, plausible futures come into view alongside probable futures. Some climate futures remain inert because they never acquire a form that decision-makers can readily think through together. Institutional familiarity can make some futures seem vivid and comfortable, so they acquire disproportionate influence despite an uncertain evidential basis. Present action is guided, in practice, by futures that become workable in thought and then workable in collective coordination. A model may describe a future in technical terms; action still depends on whether an institution can think with it, rehearse it, challenge it, and connect it to decisions. The argument developed here begins from that gap between description and use. The framework preserves evidential discipline and treats probability as essential to disciplined decision-making because it supports formal appraisal and systematic comparison; plausibility describes a distinct layer of the decision process encompassing the cognitive and institutional conditions under which a possible future becomes mentally constructible, socially shareable, culturally legitimate, and practically available for action. Under deep uncertainty, decision-makers need both formal appraisal and usable anticipation (Kay and King, 2020; Sahlin et al., 2021; Walley, 1991).

We make three contributions to climate-risk governance and the psychology of decision-making under deep uncertainty. First, it extends work on mental time travel and constructive memory from individual future simulation to institutional anticipation in climate-risk contexts (Clayton and Wilkins, 2018; Schacter et al., 2012; Suddendorf and Corballis, 2007). Second, it extends established work on processing and simulational fluency to climate-risk governance. We use simulational fluency to describe the subjective ease with which a decision-maker can assemble an uncertain future into a temporally ordered and causally connected representation that can be inspected and compared with alternatives. Our contribution lies in integrating this individual-level construct with constructive future thinking and specifying its proposed role within a multilevel framework of climate-risk scenario uptake (Alter and Oppenheimer, 2009; Mrkva et al., 2018). Third, it proposes a translational framework for climate-risk scenario practices that preserve uncertainty and make overlooked futures cognitively navigable, including those shaped by nonlinear change or genuinely unprecedented conditions (Haasnoot et al., 2013; Richter et al., 2023).

Climate-related financial risk is a central domain in which this broader cognitive problem becomes institutionally consequential as uncertain futures become available to coordinated judgement and action.

## 2 Climate risk, deep uncertainty, and scenario analysis

Climate-risk scenarios sit at the center of this problem because they are asked to perform two tasks at once. They must represent uncertain physical, economic, technological, and political pathways in forms usable by institutions constrained by shorter planning cycles and by established routines built around inherited risk categories. Climate change compresses multiple sources of difficulty into the same decision space, including long temporal horizons, nonlinear dynamics, spatial unevenness, technological transition, political conflict, and uncertainty about social response (Baer et al., 2023; Lawrence et al., 2020). Scenario analysis joins formal modeling to the cognitive and organizational work through which institutions structure uncertain futures for rehearsal and decision.

Deep-uncertainty research has developed around precisely this problem. Here, deep uncertainty refers to decisions in which the governing model and its probabilities remain contested, often alongside disagreement about valued outcomes (Lawrence et al., 2020). Radical uncertainty names the broader condition in which important futures resist exhaustive enumeration or reliable probabilistic representation (Kay and King, 2020). Lawrence et al. (2020) argue that climate decisions frequently resist optimisation over well-characterized probabilities because major uncertainties are structural as well as large. Chenet et al. (2021) argue that climate-related financial risk places pressure on disclosure-led and price-led assumptions about routine risk absorption. Kay and King (2020) make a closely related argument through the language of radical uncertainty, favoring robust preparation across multiple possible futures over confidence in spurious precision. Their phrase that “evolution is smarter than economics” condenses the intuition that adaptive systems survive through flexible preparation for uncertainties that resist conversion into risk. The value of climate-risk scenarios encompasses predictive accuracy and support for robust preparation across multiple plausible pathways (Haasnoot et al., 2013; Kay and King, 2020; Lawrence et al., 2020).

Backward-looking statistical tools that draw on historical experience face the same strain. The Green Swan report from the Bank for International Settlements argues that climate change is creating an epistemological break for financial stability analysis because nonlinear dynamics and endogenous feedbacks strain widely used methods, especially in analyses of catastrophic tail events (Bolton et al., 2020). Belloni et al. (2022) make a related point through work on euro area banks and carbon-price sensitivity. Historical data retain value, and their regularities now provide a weaker guide than conventional risk architectures tend to assume. Research in financial regulation adds that model complexity can amplify this problem by generating an appearance of precision that exceeds the available knowledge base and narrows the recognized range of plausible outcomes (Aikman et al., 2021; Bradley, 2025; Shortridge and Guikema, 2017). These concerns make scenario design a question of epistemic discipline as well as technical sophistication; its aim is to widen the space of plausible futures for structured testing under an explicit account of deep uncertainty (Aikman et al., 2021; Bolton et al., 2020; Shortridge and Guikema, 2017).

Institutional climate discourse, particularly within the financial sector, has accordingly turned toward long-horizon scenario analysis as a basis for resilience. Carney's (2015) “Tragedy of the Horizon” speech placed temporal misalignment at the center of climate-financial decision-making. More recent guidance from the Bank of England and the BIS describes scenario analysis as a key tool because climate-related financial risks exceed what standard risk models can capture, and because off-the-shelf metrics rarely provide the granular, forward-looking perspective required in practice (Bank of England, 2024; Bank for International Settlements, 2023). Firms are asked to work over longer horizons than are usual in business planning and translate spatially distant developments into local financial consequences; this requires attention to interactions between climate processes and social systems whose behavior exceeds the explanatory reach of historical analogs (Baer et al., 2023; Bank of England, 2024).

Decision-making biases enter the climate problem at the same point. Inertia can sustain previous choices even when the informational environment has changed (Kahneman, 2011; Samuelson and Zeckhauser, 1988; Alós-Ferrer et al., 2016). Psychological distance can make climate threats feel remote in time or place and abstract in their social relevance (Jones et al., 2017; Spence et al., 2012; Trope and Liberman, 2010). Attentional and perceptual biases can further influence what is noticed and how it is interpreted (Luo and Zhao, 2021; Tal and Paz, 2026). Personal experience of extreme weather can increase climate-risk perception through availability and representativeness heuristics, making hazards easier to recall and easier to compare with familiar patterns (Botzen et al., 2025). Immersive or embodied experience can heighten personal relevance and agency as well (Ahn et al., 2014). Taken together, these findings indicate that the gap between model output and timely action is partly a problem of cognitive uptake.

Mental time travel, the ability to remember the past and project into possible futures, speaks directly to this challenge (Tulving, 1985). Climate futures often feel remote because they remain experientially thin. Scenario work can begin to address that problem when it allows a form of personal pre-experience and resists an excessive initial focus on numbers. These scenario exercises can engage the same constructive capacities used in ordinary planning when they provide pathways, thresholds, actors, and situated consequences (Madore et al., 2016; McKiernan, 2017; Schacter et al., 2012). By making uncertainty inspectable and preserving the distinction between imagined futures and predictions, scenario exercises can expose hidden assumptions and trace how consequences propagate; strategic options then become easier to retrieve later in deliberation (Lee et al., 2020; McKiernan, 2017). Psychological proximity gives uncertain futures greater relevance and makes them easier to rehearse before they are lived, which can support timely personal action (Ahn et al., 2014; Jones et al., 2017).

Dynamic adaptive policy pathways reflect a similar logic, Haasnoot et al. (2013) developed pathways as a method for crafting robust decisions under deep uncertainty, and Haasnoot et al. (2024) show how a decade of applications has moved the idea into practice. A distant endpoint becomes easier to reason about once it is reworked as an unfolding route whose decision points leave room for adaptation and revision. This pathway logic provides a practical bridge between climate adaptation research and the cognitive account developed here. The challenge spans probability and representation, and its demands arise at cognitive and institutional levels, requiring climate scenarios to preserve uncertainty as they become concrete enough to support judgement. To explain this transition from formal representation to usable anticipation, we turn to research on constructive memory and Mental Time Travel.

## 3 Mental time travel and the cognitive availability of climate futures

The difficulty of acting on climate futures is partly a difficulty of mental construction. Climate scenarios often concern events that are temporally distant, spatially uneven, causally complex, and experientially thin. These features heavily tax the cognitive systems that construct possible events and organize them into sequences for comparison among futures beyond lived experience. Research on Mental Time Travel (MTT) and constructive memory provides a theoretical basis for examining why some futures become available for judgement while others remain abstract (Schacter and Addis, 2007; Schacter et al., 2012; Suddendorf and Corballis, 2007).

Findings in cognitive science provide a theoretical foundation for understanding the cognitive availability of climate futures. Remembering the past and imagining the future engage overlapping constructive and reconstructive processes, recruiting the hippocampus, medial prefrontal cortex, posterior cingulate cortex, and related regions of the default mode network, through which stored experience, semantic knowledge, current goals, and situational demands are recombined into possible events and trajectories (Addis et al., 2007; Schacter and Addis, 2007; Schacter et al., 2007, 2012; Tulving, 1985). From this perspective, memory serves as a resource for simulation, evaluation, anticipation, and preparation. Its adaptive value lies in that flexibility, and future-oriented climate judgement depends in turn on how readily a possible future can be assembled, sustained, inspected, and compared with alternatives (Pendleton and Clayton, 2026; Schacter, 2012; Schacter et al., 2012).

The concept of Mental Time Travel (MTT) marks the continuity between recollection and prospection (Suddendorf and Corballis, 2007; Tulving, 1985). Clayton and Wilkins (2018) argue that episodic memory evolved with the future in mind; Wilkins and Clayton (2019) describe the phenomenology of reconstruction, in which remembering returns the mind to a point in subjective time and plays the episode forward again, and imagining moves the mind toward a point that has never been occupied and gives events a prospective trajectory (Pendleton and Clayton, 2026). Human future thought combines computation with a phenomenological experience resembling the “remembering” of prospective events; future scenarios take the form of prospective event representations, with fragments of prior experience furnishing the structure of scenes available for advance inspection. Pendleton and Clayton (2026) place this capacity within mind time, an internally constructed and event-relative temporal framework (see also Pendleton et al., in press). Clayton et al. (in press) define transpersonal extended mental time travel (teMTT), through which individual future thinking is extended across people and generations by cultural and technological artifacts, shared narratives, communication, and durable external records (Pendleton and Clayton, 2026). For climate-risk scenarios, a future pathway requires semantic availability and a constructible form as an unfolding sequence that decision-makers can mentally enter and evaluate.

Constructive simulation can remain useful when historical experience lacks a close analog because its generative process recombines episodic elements with semantic knowledge, model outputs, current goals, and externally represented causal constraints to form novel event wholes (Addis et al., 2007; Pendleton and Clayton, 2026; Schacter and Addis, 2007; Schacter et al., 2012). The epistemological break described in climate-risk analysis weakens straightforward extrapolation from the past. Memory retains its relevance through components and organizing structures supplied by past experience. Climate science and scenario narratives extend the available relations and constraints, and durable external records keep this material available for recombination through teMTT. The resulting simulations serve as hypotheses for disciplined comparison and rehearsal, with evidential support assessed independently. Source monitoring supplies one safeguard by keeping the origins of a simulation's components inspectable. Labeling observed evidence and model outputs separately from remembered experience and imaginative construction helps preserve the distinction between coherence and evidential support (Johnson et al., 1993).

Several implications follow for climate-risk judgment, even when memory appears to concern the past alone, its functional significance lies in the way remembered material can be carried forward into present judgement and future-oriented action (Clayton et al., 2003; Clayton and Wilkins, 2018; Wilkins and Clayton, 2019). Distortion belongs to the same generative neural architecture that supports flexible simulation (Bartlett, 1932; Johnson et al., 1993; Pendleton and Clayton, 2026; Roediger and McDermott, 1995; Schacter, 1999, 2012; Schacter et al., 2011). Future-oriented judgement also draws on an interaction between rapid predictive processing and slower reflective reasoning, so candidate structures are generated quickly and then tested by asking how they relate to current goals and what consequences they imply (Evans and St, 2008; Faiella and Corazza, 2025; Schacter et al., 2012). Institutional decisions combine quantitative inputs with the cognitive processes through which people interpret and evaluate them. Decision-makers construct mental models by integrating factual knowledge and numerical outputs with remembered experience and prior beliefs (Johnson-Laird, 1983). This integration makes the interpretation of model outputs sensitive to the representational resources already available to the decision-maker. Some model outputs may have limited uptake when their semantic input lacks the episodic scaffolding required to make the future feel personally or institutionally plausible.

Comparative work broadens the frame by locating future-relevant memory across human and non-human species and establishing the evolutionary level of the framework. Clayton and Dickinson (1998) introduced the term episodic-like memory to refer to the behavioral components of episodic memory in the absence of any assumed accompanying elements of phenomenological consciousness of episodic recall, to enable tests of such abilities in non-verbal animals, initiated through research on the mnemonic abilities of scrub-jays to remember what happened, where it happened, and when it occurred (Clayton et al., 2000). Clayton et al. (2003) extended the argument into future planning, and later work broadened the discussion to apes and cephalopods. Evidence includes tool saving for future use in apes and corvids, with varying evidential strength across studies (Mulcahy and Call, 2006; Boeckle et al., 2020); planning for future breakfasts in corvids (Raby et al., 2007); flexible and future-oriented foraging cognition in cuttlefish (Billard et al., 2020); and delayed gratification in cuttlefish (Schnell et al., 2021b). Pendleton and Clayton (2026) place these distantly related taxa within a wider comparative discussion of temporal cognition. Tulving's phenomenological criteria, including autonoesis (the awareness of oneself as the author and owner of a memory) and chronesthesia (the subjective awareness of the passage of time), remain difficult to assess in non-linguistic subjects (Griffiths et al., 1999; Tulving, 1985, 2002). Even so, behavioral evidence indicates that flexible, future-relevant memory has emerged in distantly related lineages. The appearance of these capacities in separate lineages may reflect convergent evolution where distantly related organisms faced similar selection pressures, or independent evolution where those pressures differed (Schnell et al., 2021a,b; Schnell and Clayton, 2021). In both cases, selection favored systems that allowed organisms to prepare for multiple eventualities before the moment of encounter. Modern organizations use scenario analysis for a functionally analogous anticipatory task. Their decision routines distribute the associated reasoning across language and external representations.

Experimental work on episodic specificity induction fits this account closely because brief cueing procedures can alter the detail and structure of later remembering and imagining. Madore et al. (2014) found that specificity induction increased internal detail generation in memory and imagination tasks, and Madore and Schacter (2016) showed that this selectivity persisted in comparison tasks requiring generative search. Subsequent work interprets the mechanism in terms of event construction (Madore et al., 2019, 2016). These studies arise outside climate governance and strengthen the broader claim that future-oriented judgement depends on how coherent scenes are assembled and maintained. For climate-risk scenarios, this evidence locates exercise design within modeling itself. Design choices affect the richness and coherence of constructed futures as well as their later retrieval in deliberation (Madore et al., 2019, 2016; McKiernan, 2017).

With that perspective in place, the practical role of abstraction becomes easier to see. Numbers and models influence behavior when they enter a usable mental form, whether as a pathway, a threshold, a scene, or a sequence that people can inspect and compare (see Table 1 below). Climate research already points in this direction. Lee et al. (2020), for example, find that episodic future thinking about climate-related risks can increase both perceived risk and pro-environmental behavior. Keller et al. (2022) find that psychological distance in climate cognition is multidimensional and context-sensitive, while van Valkengoed et al. (2023) argue that distance has often been overstated as a master explanation for climate inaction. These findings position cognitive workability as a candidate contributor to scenario uptake. Clear construction supports inspection and comparison, creating one condition under which a future may guide action.

**Table 1:** Operationalizing usable mental formats in climate-risk governance.

Mental format		Translation of abstract input	Illustrative governance use	Evidential safeguard
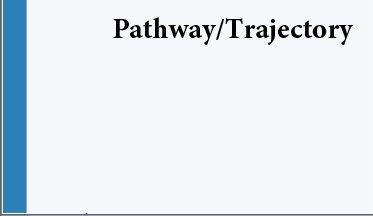		Links drivers to exposures, vulnerabilities, consequences, feedbacks, and decision points.	A bank traces a transition or flood shock through counterparties, collateral, liquidity, and management responses; a ministry maps an adaptation pathway across sequenced decisions.	Display alternative causal chains; identify uncertain links; test sensitivities; retain branch points.
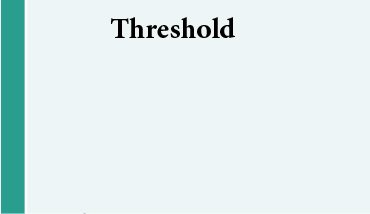		Turns a value or range into a provisional signpost, trigger, or condition for changed action.	A risk committee sets an escalation range for portfolio review; an adaptation plan links observable signposts to staged contingency measures.	Represent threshold uncertainty with ranges. Cite the evidence; specify monitoring and updating rules.
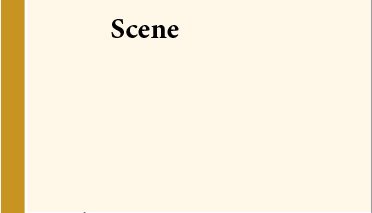		Situates actors, place, time, constraints, and operational consequences in a concrete decision context.	A tabletop or reverse stress test asks participants to navigate a locally specific disruption, including distributional and cross-functional consequences.	Mark illustrative details; distinguish evidence from narrative completion; compare multiple scenes.
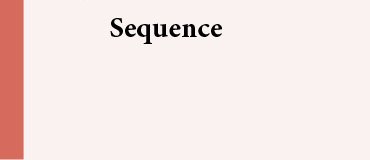		Orders intermediate events, delays, interactions, cascading effects, and possible reversals.	A stress-test timeline or contingency playbook follows how impacts and responses unfold across functions and decision horizons.	Vary timings and orderings; show alternative branches; document assumptions; revise after rehearsal.

Illustrative translations from abstract scenario inputs to inspectable and revisable governance practices. These complementary design options can be combined in varied sequences. Their purpose is to keep assumptions and consequences inspectable while preserving the distinction between plausibility and probability.

Table 1 translates these four mental formats into illustrative governance applications and associated evidential safeguards.

## 4 A cognitive architecture of plausible climate futures

The framework proposed here addresses the question of why some climate futures become action-guiding and others remain inert. Plausibility refers here to the degree to which a possible future can be mentally constructed, narratively organized, socially stabilized, and culturally legitimated as a candidate for action, while probability remains important because future-oriented judgement still depends on formal appraisal as well as cognitive and social uptake (Addis and Szpunar, 2024; Chenet et al., 2021; Kashima et al., 2025; Paddeu and Lyons, 2024; Pisor et al., 2023). Plausibility tracks a different layer of explanation, one concerned with why some modeled futures become usable in thought and coordination while others remain behaviorally weak (Kashima et al., 2025; Schacter et al., 2012).

Constructive simulation forms the first component of this architecture. Constructive simulation supports the forms of prospective inspection and comparison developed in this framework. Recognizable sequences within familiar scripts draw on established semantic scaffolds and are often easier to construct; unprecedented and highly nonlinear futures place heavier demands on imagination (Clayton and Wilkins, 2018; Schacter et al., 2012). Narrative scaffolding forms the second component. A causally connected temporal sequence gives thought something to move through. A quantitative projection or distant target may be conceptually intelligible and difficult to inhabit. Pathways trace the development of conditions over time; thresholds mark changes in their course and in the accumulation of consequences. Scenario work often proves valuable because it furnishes that path through uncertainty (Paddeu and Lyons, 2024).

Social stabilization forms the third component. Futures gain traction through discussion, rehearsal, routines, planning exercises, public discourse, and shared representations. In this respect, future-oriented cognition extends beyond the solitary mind and enters collective life. Hazan et al. (2024) and Kashima et al. (2025) argue that collective anticipation shapes identity and cooperation, which in turn influence action. Here, social stabilization occurs when repeated use embeds a contested future within organizational routines, and disagreement may persist within that shared representation. Cross-functional scenario workshops can jointly map causal pathways and decision points and preserve a record of agreement and unresolved disagreement. Repeated tabletop or stress-testing exercises can return to that record. A structured audit can establish the scope and unresolved assumptions of the scenario process, then follow the resulting scenarios into deliberation and decision-making (Cordova-Pozo and Rouwette, 2023; Oliver, 2023; Paddeu and Lyons, 2024; Reed et al., 2013).

Cultural legitimation forms the fourth component. A future also has to become legible within a cultural world. Identity, local history, lived values, and inherited temporal orientations influence what a community or institution treats as realistic, responsible, threatening, or normal (Adger et al., 2013; O'Reilly et al., 2020; Pisor et al., 2023). Plausibility depends on reception as well as representation. Building on research on processing and simulational fluency, we use simulational fluency to describe the experienced ease with which an individual constructs and maintains a possible future as an ordered event representation from presented information and prior knowledge (Alter and Oppenheimer, 2009; Mrkva et al., 2018). Here, cognitive availability describes whether a constructed future can be retrieved and used during comparison or deliberation. Simulational fluency concerns the experienced ease of construction; the availability heuristic concerns judgements shaped by ease of recall. Simulational fluency differs from general processing fluency, which concerns ease of processing the presentation itself; vividness, which concerns perceptual or phenomenological richness; episodic specificity, which concerns event detail; narrative coherence, which concerns structural relations among scenario elements; and institutional uptake, which is a downstream collective outcome. It also differs from evidential plausibility because simulational ease and evidential support can diverge; we propose the candidate relation that scenario form may influence judged plausibility partly through simulational fluency.

The psychology of magic provides a mechanistically informative illustration of how prediction, attention, expectation, inference, and reconstructive memory interact under controlled violations of expectation. These effects show that coherent priors can preserve an incorrect interpretation by suppressing anomalous information. Clayton and Wilkins (2017) used magic in a science-arts exploration of memory and mental time travel; Garcia-Pelegrin and colleagues extended that programme across humans and non-human animals, using magic effects to probe mental time travel, expectation, deception, and perceptual inference in comparative perspective (Garcia-Pelegrin et al., 2020, 2024, 2022). Illusions are useful here because they make cognitive blind spots visible. An audience can miss a sleight-of-hand effect because perception follows the expected sequence, a well-studied result in the psychology of magic (Kuhn et al., 2008; Pailhès and Kuhn, 2023). In the framework proposed here, this work supplies an analogy and a testable hypothesis for climate governance, where familiar scenario architectures may direct attention away from discontinuities.

## 5 Narrative, vividness, and the uses and limits of pre-experience

Narrative changes the cognitive handling of climate futures by arranging possible developments in temporal order. That claim fits a long tradition in which narrative is treated as a way of constructing reality and integrating new material into existing interpretive frames (Bruner, 1991; Chong and Druckman, 2007). In climate-focused work, Richter et al. (2023) argue that scenario development often underuses knowledge of how cognitive and emotional processes shape communication. Coulter et al. (2019) connect climate adaptation narratives with future thinking and practical planning. More recent work on scenario narratives in the Shared Socioeconomic Pathways indicates that narrative structure and quantified modeling can illuminate different features of the same future space (Xexakis et al., 2026). Within this framework, narrative scaffolding gives possible futures a form that thought can enter and move through. That role extends beyond presentation to shape the comparison and recall of futures and to reveal latent assumptions.

Vivid pre-experience can sharpen strategic engagement. Neef et al. (2023) find that optimistic narrative future visions can influence emotion and behavioral intention, and Wenzel et al. (2024) find that imagined positive futures can increase collective-action commitment through hope. Temporally ordered narratives are especially useful when future outcomes depart sharply from incremental continuation. A pathway reframes an abstract endpoint as a continuous temporal sequence that helps people navigate breaks in continuity and follow the cascading effects of crossed thresholds. When a scenario is built in this way, it becomes more compatible with episodic processing and more available for later retrieval in deliberation (Schacter et al., 2012). Affect and imagery are part of that process. Risk perception extends beyond calculation alone; consistent with the affect heuristic, climate judgements are strongly influenced by the images and feelings attached to possible futures (Leiserowitz, 2006; Slovic et al., 2004).

These points also clarify the role of qualitative scenario methods. Quantitative approaches are frequently prioritized by financial stakeholders seeking concrete metrics, rendering qualitative approaches seemingly less decision-useful by comparison (Ding et al., 2025). Supervisory and disclosure frameworks have sometimes implied a hierarchy in which narrative approaches appear preliminary and quantitative approaches appear more advanced (Task Force on Climate-related Financial Disclosures, 2017). Qualitative methods align closely with the cognitive mechanisms discussed here and support plausibility by bringing scenarios closer to stakeholders' experience. Developing them with intended users can also uncover assumptions embedded in the scenario (Reed et al., 2013). Participatory scenario development gives users a direct role in framing the problem and helps future narratives travel across institutional settings.

Qualitative and quantitative approaches function most effectively as complementary components of the same anticipatory process. Integrated approaches can translate narratives into structured modeling inputs and retain the contextual richness that gives scenarios practical force (Booth et al., 2016; Hirsch et al., 2013; Mallampalli et al., 2016). Narrative and quantitative analysis can also be combined more explicitly in climate futures work, as recent research on SSP narratives demonstrates (Xexakis et al., 2026). A clear storyline can constrain what is quantified, and quantitative analysis can test the implications and tensions within the storyline.

The same mechanisms also carry an epistemic risk because vividness and truth are different properties. Green and Brock (2000) find that transportation into narrative can influence belief, while Unkelbach and Rom (2017) find that ease of processing can influence perceived truth. Troy et al. (2024) find that positive and negative depictions of climate futures can affect action intentions through distinct psychological routes. Evidential support can make a future convincing; repetition and fluent imagining can confer a separate sense of coherence. This conditional relation between vividness and evidence is why scenario work requires careful design.

Questions of quantification enter here as well. Quantification brings disciplined and explicit comparison to climate-risk decision-making. Its practical value depends on the quality of the scenario structure to which it is attached. An IFRS Foundation staff paper reproduces TCFD guidance identifying a clear narrative of drivers, pathways, constraints, and outcomes as a prerequisite for effective quantification and warning that a rush to quantification “may serve only to put an ever-finer point on an ineffective tool” (IFRS Foundation, 2023). Numbers attached to a weak scenario can create the appearance of control and leave understanding unchanged; numbers attached to an intelligible pathway can deepen analysis and make comparison more rigorous. The central issue concerns sequencing, with scenario narrative and quantification working best when they develop together in a mutually constraining way.

## 6 Institutions, culture, and scenario uptake

Climate scenarios enter organizations with existing mandates, professional norms, reporting cycles, risk categories, political constraints, and shared assumptions about what counts as realistic. A technically intelligible scenario may remain institutionally weak when it falls outside the interpretive routines through which decisions are made, illustrating the social as well as analytical character of scenario uptake.

The extension from persons to institutions occurs through distributed coordination of prospective representations across people, artifacts, routines, and decision rights; autonoetic phenomenology remains an individual-level property. In the terms used here, teMTT supplies the bridge through which individuals coordinate prospective representations through narratives, models, records, communication, meetings, routines, and decision rights. Articulation and comparison bring individual simulations into collective deliberation, where repeated strategic conversation may stabilize shared representations. Mandates, professional norms, authority, incentives, and culture then shape institutional contestation and determine which representations are retained or excluded. Institutional uptake in this sense is an emergent and contingent outcome shaped by interactions across levels; the fluency experienced by any one decision-maker supplies one influence among several. Institutional routines reshape individual priors, which then guide attention within institutionally permissible frames. This interaction creates feedback across levels.

Future-oriented judgement scales from persons to groups and institutions; personal prospection and collective anticipation share some processes and remain distinct, with institutions transforming private expectations through collective deliberation, authority, routines, and culture. Hazan et al. (2024) find that personal future thought and collective future thought are related yet distinct, while Kashima et al. (2025) argue that collective future thinking influences cultural dynamics. Institutions set the bounds of what futures can be discussed and what uncertainties can be recognized. Those bounds determine which pathways gain enough shared recognition to guide coordinated action.

Institutions regulate what may be discussed, so social stabilization can reproduce blind spots established by professional norms and reinforced by political culture. Scenario governance should record which pathways, assumptions, stakeholders, and forms of evidence are excluded; preserve reasoned dissent and minority scenarios; broaden or rotate participation; and periodically subject scenario sets and decision rules to independent challenge. These safeguards make exclusion visible and contestable and leave institutional neutrality as an ongoing governance challenge.

Scenario planning belongs within that broader process. Paddeu and Lyons (2024) argue that foresight gains force when shared mental models of the present system are built before scenarios are developed, and Cordova-Pozo and Rouwette (2023) find that the effectiveness of scenario planning depends heavily on process design. Oliver (2023) argues in similar terms that scenario planning becomes useful through strategic conversation and actionable knowledge. Documentation alone is seldom sufficient to shift organizational judgement. Repeated rehearsal changes the common frame as participants compare and interpret future possibilities, a process central to organizational sensemaking (Weick, 1995).

This institutional perspective also explains why climate-risk scenario analysis should be treated as one element within a wider organizational capability. Its value extends beyond analytical refinement into product and service design, governance, training, and professional development. Riedy and Waddock (2022) argue that sustainable transformation requires shared social imaginaries, while Chevrier et al. (2025) argue that collective positive prospection can motivate social change through achievability, desirability, criticism of the status quo, and level of abstraction. Koch et al. (2025) argue that futures consciousness, the human capacity to understand, anticipate, and intentionally prepare for future trajectories, is a capability that can be learned and assessed. Taken together, these literatures support the practical conclusion that organizations need capacity for anticipation, interpretation, comparison, and revision alongside scenario production.

A longer view of scenario practice points in the same direction. Pierre Wack, reflecting on early Shell scenario work, argued that the task was to influence the “microcosms” through which decision-makers pictured reality, since a scenario package alone would be “like water on a stone” without that shift in mental image (Wack, 1985). That observation anticipates the challenge developed in this article. Scenario work succeeds when it changes how futures are imagined and discussed inside institutions; adding another report to the archive seldom achieves that shift.

Culture enters this capacity at every stage. Climate anthropology and adaptation research indicate that futures are received, translated, authorized, and contested through place, identity, seasonal rhythm, and local knowledge (Adger et al., 2013; Bremer and Schneider, 2024; de Wit and Haines, 2022; Kassam et al., 2021; O'Reilly et al., 2020; Pisor et al., 2023). Plausibility varies across social worlds. The same scenario may travel well in one institutional setting and poorly in another because temporal frameworks and moral commitments differ. A culturally adequate account of foresight must explain that variation as a substantive feature.

Cultural legitimacy and scientific validity answer different questions. Scientific evidence constrains claims about physical processes and supportable outcomes. Local knowledge informs interpretations of exposure, and identities and moral commitments shape the acceptability and feasibility of a response. Together, these local factors affect whether an institution or community can act on a pathway (Adger et al., 2013; Pisor et al., 2023; Reed et al., 2013). When these sources appear to conflict, scenario practice should distinguish empirical from normative disagreement and document the assumptions at issue; unresolved alternatives should stay available for comparison until the grounds for each have been examined. Cultural uptake leaves the evidential status of an unsupported biophysical claim unchanged; the completeness of a technically sound global pathway also depends on local conditions that shape distribution and implementation.

## 7 Quantification, formal methods, and disciplined judgement

A cognitive account of plausibility combines cognitive usability with evidential discipline in climate-risk judgement. By making assumptions explicit, formal methods keep comparison disciplined and anchored in evidence. Their value is greatest when they are applied to pathways whose causal structure has already been made clear.

Formal methods still have an important role within this broader account. Belief-function approaches such as Dempster–Shafer theory preserve distinctions among belief, plausibility, and uncertainty when evidence is incomplete (Shafer, 1976). Plausibility has a formal meaning within Dempster–Shafer theory that is distinct from the cognitive and institutional sense developed in this article. The former is a property of an evidential representation; the latter concerns the conditions under which a possible future becomes available for judgement and coordinated action. Reviews of decision-making with belief functions indicate that such approaches can represent ignorance and incomparability more explicitly than standard expected-utility treatments permit (Denoeux, 2019). Robust Bayesian and imprecise Bayesian approaches can likewise support disciplined updating while keeping uncertainty about priors, utilities, and model structure in view (Sahlin et al., 2021; Walley, 1991). More recent work comparing imprecise Bayesianism and Dempster–Shafer theory under ambiguity indicates that the choice among these frameworks influences how automated or formalized decisions respond to missing information (Radzvilas et al., 2025). Applied work also indicates that Dempster–Shafer and Bayesian tools can be combined in practice when decision environments contain interacting uncertainties and multiple pathways of risk transmission (Fan et al., 2025).

These approaches keep judgement within the process while making its assumptions more explicit and open to revision. Belief-function approaches can retain divergent expert views by operating over sets of plausible future states and delaying compression into a single estimate, thereby maintaining adverse and disruptive futures through the quantification stage.

A broad view of plausible futures aligns directly with this point. Radical uncertainty sets limits on the compression of uncertainty into single estimates, so decision systems need breadth as well as precision. Breadth allows neglected futures to enter deliberation, and precision helps organizations compare implications once those futures are on the table. Human-oriented approaches and formal methods can work together. Narrative pathways, simulational fluency, belief functions, Bayesian updating, organizational rehearsal, and integrated qualitative-quantitative analysis each contribute to disciplined anticipation when they are combined carefully.

Systematic search across a wider set of pathways may improve institutional comparison and stress testing, provided that inputs, assumptions, uncertainty, and provenance remain inspectable. Such systems remain decision-support tools, with human institutions retaining responsibility for problem framing, evidential judgement, interpretation of distributional and moral consequences, accountability, and the decision itself.

Several design implications follow. Future-oriented work gains strength from causal pathways that guide subsequent quantification and improve the cognitive navigability of neglected futures. Organizations must also cultivate revisability and epistemic humility. Scenario exercises gain further strength as elements of routine governance when training supports interpretation across functional boundaries. Within that kind of system, quantification supports judgement because it grows out of an intelligible pathway, and narrative supports judgement because it remains anchored to evidence while keeping uncertainty in view.

## 8 Discussion—A cognitive framework of plausible climate futures

**Figure 1:**
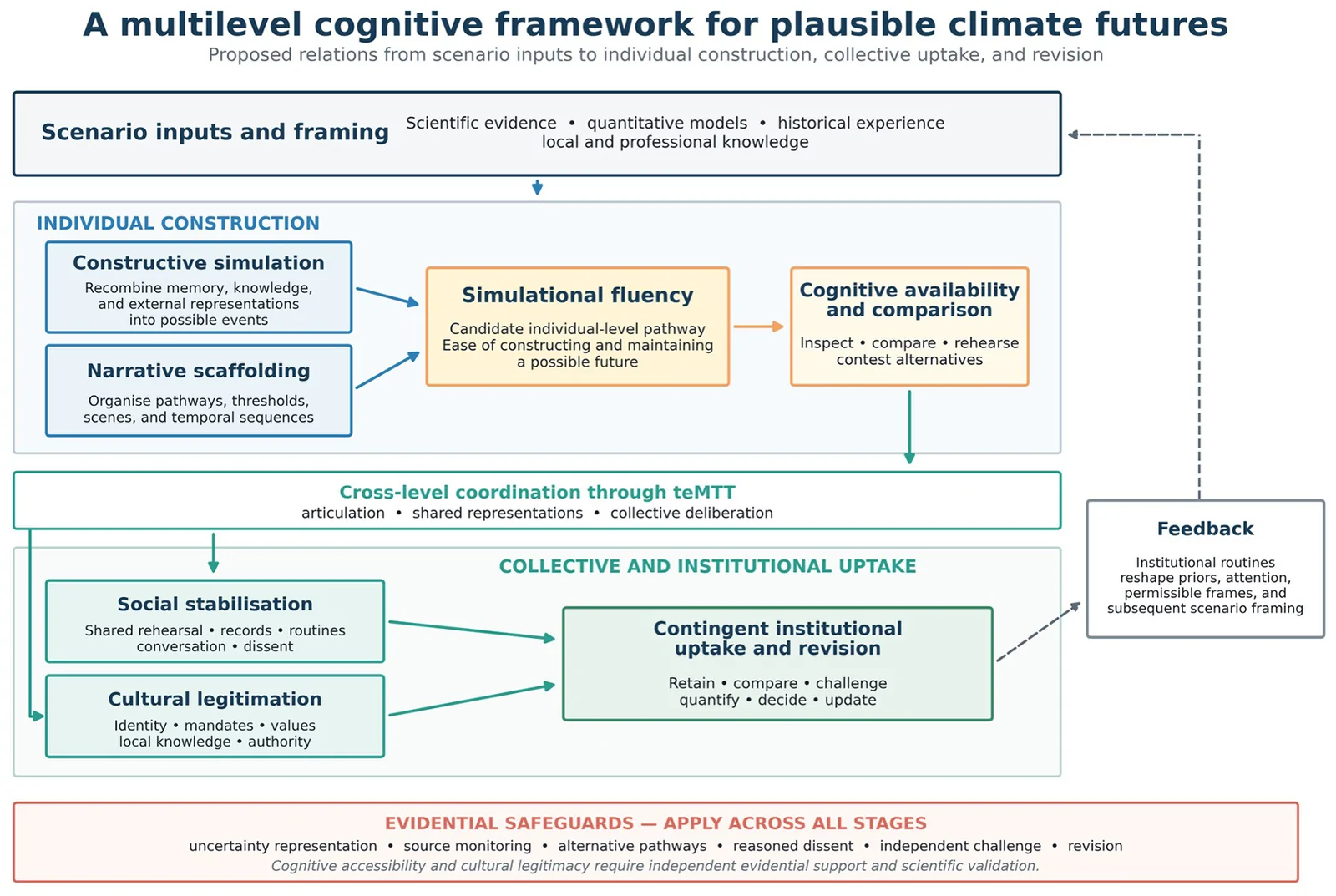
A multilevel cognitive framework for plausible climate futures. Scenario inputs are organized through constructive simulation and narrative scaffolding; simulational fluency is presented as a candidate individual-level pathway to cognitive availability and comparison. Cross-level coordination through teMTT supports social stabilization and cultural legitimation, which contribute to contingent institutional uptake and revision. Feedback reshapes subsequent scenario framing, and evidential safeguards apply across all stages. Arrows represent proposed relations for empirical testing.

The framework (see Figure 1 below) developed here treats climate-risk scenario analysis as a cognitive and institutional practice. For an uncertain climate future to guide present action, it must move from probabilistic abstraction into a form that decision-makers can construct, navigate, discuss, contest, quantify, and revise. The framework proposes four interacting conditions that may support this transition, comprising constructive simulation, which integrates abstract information into event-based mental representations that decision-makers can inspect and pre-experience; narrative scaffolding, which provides the temporal sequences and causal structure needed to navigate thresholds, nonlinear changes, and cascading effects; social stabilization, through which repeated collective rehearsal and strategic conversation may move individual prospection into shared representations; and cultural legitimation, which influences whether scenarios remain legible within the identities, values, mandates, and local knowledge of the receiving institution.

Across these stages, simulational fluency is proposed as one candidate individual-level pathway. Mathematical probability contributes to formal appraisal; the ease of constructing and maintaining a scenario may influence its cognitive availability. Recognizing this proposed pathway suggests that organizations can test whether alternative exercise designs make neglected or unprecedented futures easier to construct and retain, and whether this reduces bias toward familiar, incremental trajectories.

In practical terms, the framework places causal pathway construction before intensive quantification, after which narrative and numerical analysis should develop iteratively and constrain one another. Belief-function approaches and Bayesian updating can then test those pathways under explicit safeguards against false precision (Denoeux, 2019; Sahlin et al., 2021; Shafer, 1976; Walley, 1991). This integrated approach positions formal models as scaffolds for organizational sensemaking and preserves human judgement.

Several design principles follow. Scenario exercises should build causal pathways before intensive quantification; make thresholds, feedbacks, and transmission mechanisms explicit; include enough contextual detail for decision-makers to mentally navigate the pathway; test whether neglected futures remain cognitively available during later deliberation; involve users early so scenarios become socially shared; and preserve multiple plausible futures together with the uncertainty they represent.

A science of plausible climate futures asks how uncertain trajectories become thinkable enough to matter before they are lived. By bridging cognitive psychology, comparative cognition, scenario planning, and climate-risk governance, the framework developed here offers a structured approach grounded in the fundamental evolutionary premise that memory evolved with the future in mind. Navigating the radical uncertainty of climate change depends on precise calculation and the capacity of minds and institutions to inhabit possible futures cognitively.

### 8.1 Scope, boundary conditions, and future research

This Perspective develops an integrative framework from established findings in constructive memory, mental time travel, processing and simulational fluency, scenario practice, and organizational sensemaking. Its theoretical and translational contribution specifies relationships among these literatures and derives design implications for climate-risk governance. Building on well-established component literatures, the proposed relations define an agenda for empirically testing and refining their integration within climate-risk institutions.

Formal scenario infrastructures in climate-related finance must address nonlinear risk over unusually long horizons; these conditions make the cognitive problem especially visible and establish finance as the principal applied setting. Comparative work can examine the framework's transfer beyond finance across domains of deep uncertainty, including public adaptation and local governance. Future research can manipulate causal sequencing and contextual detail within scenario exercises, then examine how collective rehearsal and evidential safeguards alter later access and use. Simulational fluency can be assessed through reported construction ease and construction latency, with vividness and narrative coherence modeled separately. Such work would clarify boundary conditions while preserving the framework's present value as a conceptual synthesis and design guide.

## Data Availability

The original contributions presented in the study are included in the article/supplementary material, further inquiries can be directed to the corresponding author.
